# Smoking and endometriosis: A narrative review

**DOI:** 10.18332/tid/203429

**Published:** 2025-06-26

**Authors:** Alexandre Vallée, Anis Feki, Loic Josseran, Jean-Marc Ayoubi

**Affiliations:** 1 Department of Epidemiology and Public Health, Foch Hospital, Paris, France; 2 Department of Gynecology and Obstetrics, University Hospital of Fribourg, Fribourg, Switzerland; 3 Département Hospitalier d'Epidémiologie et de Santé Publique, Hôpital Raymond-Poincaré, Paris, France; 4 Centre de Recherche en Epidémiologie et Santé des Populations, Université de Versailles-Saint-Quentin-en-Yvelines, Université Paris-Saclay, Paris, France; 5 Medical School, University of Versailles Saint-Quentin-en-Yvelines (UVSQ), Versailles, France; 6 Department of Obstetrics, Gynecology and Reproductive Medicine, Foch Hospital, Paris, France

**Keywords:** tobacco smoking, endometriosis, inflammation, oxidative stress, DNA damage

## Abstract

Endometriosis is a chronic gynecological disorder affecting 6–10% of women of reproductive age. While its etiology is multifactorial, growing evidence suggests that tobacco smoking may contribute to its development and progression through inflammatory, oxidative, and hormonal mechanisms. This narrative review examines the relationship between tobacco smoking and endometriosis, integrating molecular insights to clarify potential biological pathways and highlight areas for future research. A search was conducted in PubMed/MEDLINE and Web of Science, including only original research articles published in English. Studies on both human and animal models were considered, without restrictions on participant age, to provide a comprehensive overview of tobacco-related mechanisms in endometriosis. Tobacco smoke components, such as nicotine and polycyclic aromatic hydrocarbons, can initiate and sustain an inflammatory response, leading to the release of pro-inflammatory cytokines and recruitment of immune cells. Tobacco smoking also induces oxidative stress, disrupting cellular functions and damaging DNA. Moreover, it can cause hormonal dysregulation and interfere with hormone-related signaling pathways. Epigenetic modifications, including DNA methylation and histone modifications, can also be induced by tobacco smoking. These changes affect the expression of genes involved in inflammation, cell proliferation, and hormone signaling, contributing to the pathogenesis of endometriosis. Future research should prioritize longitudinal studies with objective biomarkers to strengthen causal inference. Studies integrating omics approaches can further clarify tobacco-induced molecular alterations in endometriosis. Public health policies should incorporate targeted smoking prevention and cessation programs for women at risk.

## INTRODUCTION

Endometriosis is a chronic gynecological disorder characterized by the abnormal growth of endometrial-like tissue outside the uterus; it affects 6–10% of women of reproductive age^1,2^. This condition affects millions of women worldwide and can cause significant pain, infertility, and other debilitating symptoms^3,4^. Biologically, endometriosis is an estrogen-dependent, chronic, and inflammatory gynecological disease that is defined by the proliferation of functional endometrial tissue developing outside the uterine cavity^5^. The available evidence suggests that the development of endometriosis is characterized by a complex interplay of various factors. While the exact causes of endometriosis remain elusive, researchers have explored various factors that may contribute to its development and progression. One area of interest in understanding endometriosis is the impact of environmental factors on the disease^6^. Among these factors, the association between endometriosis and tobacco smoking has gained attention^7-9^. Tobacco use, in various forms such as cigarette smoking, cigar smoking, or smokeless tobacco products, is known to have detrimental effects on human health, contributing to numerous diseases, including cardiovascular disorders, respiratory conditions, and various types of cancers^10^. However, the connection between tobacco smoking and endometriosis has been a subject of debate and investigation^9^. Thus, understanding the relationship between endometriosis and tobacco smoking is crucial for several reasons. First, endometriosis affects a significant number of women globally, and identifying modifiable risk factors can help in preventive efforts. Second, establishing a clear connection would emphasize the importance of smoking cessation interventions and raise awareness among healthcare providers and affected individuals about the potential risks associated with tobacco use. Lastly, unravelling the underlying mechanisms could pave the way for targeted therapeutic strategies to mitigate the impact of smoking on endometriosis and improve patient outcomes.

By examining the possible molecular and cellular mechanisms through which tobacco smoking may contribute to endometriosis, we can gain valuable insights into the impact of environmental exposures on this complex and often debilitating condition. Thus, this review aims to delve into the molecular understanding of the relationship between endometriosis and tobacco smoking.

PubMed/MEDLINE and Web of Science databases were used for the search, with only articles in English language, using the following terms: ‘endometriosis’, ‘tobacco’, ‘tobacco smoking’, ‘inflammation’, ‘oxidative stress’, ‘hormonal dysregulation’, ‘DNA damage’, ‘immune dysfunction’, and ‘angiogenesis’. The search strategy is provided in the Supplementary file. Two authors performed the strategy research (AV and JMA), and three authors performed the selection of the articles (AV, AF and JMA). No restriction was made for selection of studies concerning animals and humans, and also age of women^11^. Only original research articles were included in this review to provide information about the association between tobacco and endometriosis. Literature was searched from inception to December 2024. Based on the 4358 articles, 44 original articles were included in the narrative review.

## SMOKING AND ENDOMETRIOSIS

### Overview of tobacco and endometriosis

Several environmental factors, including reproductive, lifestyle and behavioral factors, have been linked to the etiology of endometriosis; however, the association with some of these factors remains inconclusive^9,12-14^ . Many recent studies have reported and association between tobacco smoking and increased risk in endometriosis^15-17^, whereas the authors of the latest meta-analysis published in 2014 concluded that there was no association between smoking and endometriosis ^9^. Nevertheless, the majority of the studies included in that meta-analysis were based on self-reports and provided crude estimates of association^9^. In contrast, a recent study, focused on more than 2 million women, has shown that women with both a family history of smoking and smoking themselves have higher risk of endometriosis than the general population (incidence rate ratio, IRR=4.28; 95% CI: 2.43–7.55)^16^. Among more than 500000 women, heavy tobacco users compared with never users presented a higher risk of endometriosis (summary relative risk=1.35; 95% CI: 1.15–1.59)^18^. Moreover, exposure to secondhand smoke during childhood due to maternal smoking was associated with increased odds of an endometriosis diagnosis (OR=2.70; 95% CI: 1.11–6.60)^17^.

Tobacco use encompasses smoking cigarettes, cigars, or pipes, as well as consuming smokeless tobacco products. The harmful effects of tobacco on human health are well documented, particularly its association with cardiovascular diseases^19^, respiratory conditions, and various cancers^20^. However, the impact of tobacco on gynecological disorders like endometriosis is less widely known.

### Molecular pathways involving tobacco smoking and leading to endometriosis

**Figure 1:**
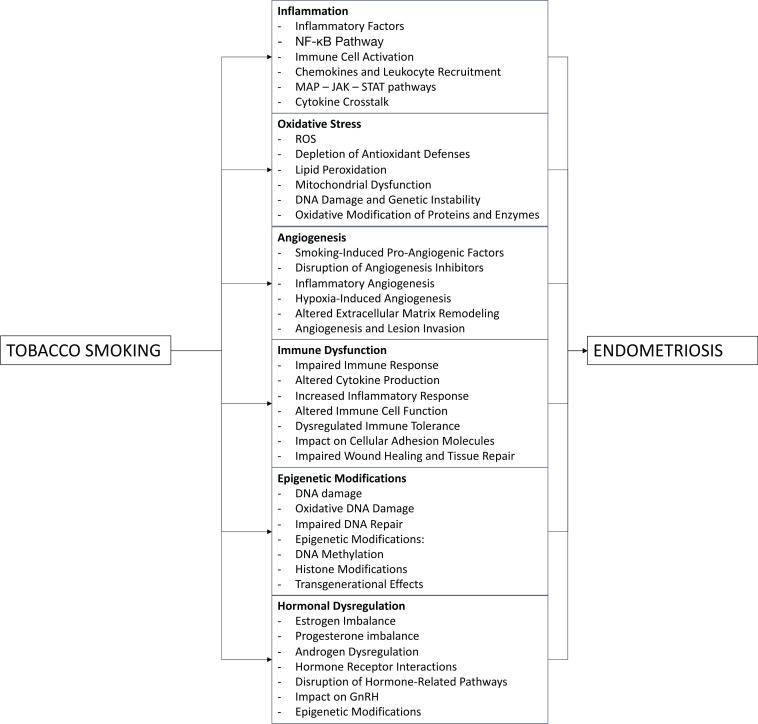
Molecular and cellular mechanisms involved in smoking-induced endometriosis

The association between tobacco smoking and endometriosis involves complex molecular pathways that contribute to the pathogenesis of the disease. Here, we provide detailed insights into the molecular pathways that connect tobacco smoking and endometriosis (Figure 1).

### Inflammation

Tobacco smoke contains a wide array of toxic compounds that can initiate and sustain an inflammatory response in the body^21-23^ (Table 1). These compounds include nicotine, carbon monoxide, polycyclic aromatic hydrocarbons (PAHs), and volatile organic compounds (VOCs)^24-26^. When inhaled, these substances can directly activate immune cells and stimulate the release of pro-inflammatory cytokines, chemokines, and growth factors^21^.

**Table 1:** Characteristics of the original articles selected for the review

*Molecular pathway*	*Ref.*	*Year*	*Objective*	*Study type*	*Type*	*Human/animal*	*Sample*	*Experimental technique*	*Findings*	*Markers*
**Inflammation**	[23]	2006	To investigate the molecular mechanisms of inflammatory responses caused by cigarette smoke extract	*In vitro*	Cell line study	Human monocytic cell line (mature monocytes) MonoMac6	NR	Western blotting, immuno-precipitation and posttranslational modifications; Electrophoretic Mobility Shift Assay; ELISA for IL-8 and TNF-alpha	Cigarette smoke-induced release of IL-8 is associated with activation of NF-B via IKK and reduction in HDAC levels/activity in macrophages	IL-8 and TNF-alpha, histone deacetylase (HDAC) activity, HDAC1, HDAC2, and HDAC3 protein levels
	[24]	2005	To provide new data for ‘tar’ and nicotine using an updated	Observational	Human study	Volunteer smokers	52	Calibrated electrochemical CO analyzer (Monoxor II, Bacharach Inc.)	Increased quantities of PAH and CO among smokers	PAHs and CO
	[25]	2020	To determine and describe groups with distinct exposure profiles	Observational	Population-based	Human	6724	Concentrations of a set of urinary tobacco biomarkers	Heterogeneity in urinary biomarkers of exposure to nicotine, TSNAs, VOCs, and PAHs	Exposure to nicotine, TSNAs, VOCs, and PAH
	[32]	2008	To investigate the effects of tobacco smoke on apoptosis induction and NF-κB signaling modulation with the goal of understanding tobacco smoke- associated disease pathogenesis	Animal	Rat model	Rats	NR	Western blot analyses; Electrophoretic mobility shift assay (EMSA)	Tobacco smoke resulted in inhibition of NF-κB activity, noted by suppression of inhibitor of κB (IκB) kinase (IKK), accumulation of IκBα, decrease of NF-κB DNA binding activity, and downregulation of NF-κB - dependent anti-apoptotic proteins, including Bcl-2, Bcl-xl, and inhibitors of apoptosis	NF-κB p65, p50, IκBα, IκBβ, HSP70, Bcl-2, Bcl-xl, c-IAP1, c-IAP2, XIAP, p53, Bax, caspase 8, caspase 9, caspase 3 and actin
	[39]	2019	To determine that serum chemokines and MMPs will be altered in women with endometriosis compared to women without disease	Observational	Case-control		41	Multiplex cytokine immunoassay	Chemokines (CCL1, CCL22, and CCL11) and cytokine (IL-10) are elevated in endometriosis cases	Chemokines and cytokines
	[40]	2018	To model the inflammatory microenvironment in endometriotic lesions	*In vitro*	Monocyte cell model	THP-1 human monocyte cell line (TIB-202)	NR	Quantitative real-time PCR analysis	Niclosamide inhibits macrophage-dependent endometriotic epithelial cell viability and production of cytokines and chemokines in treated cells through STAT3 and/or NFKB signaling	NFKB and STAT3
	[43]	2018	To develop an *in vitro* screening panel to identify whether flavorings added to tobacco products	*In vitro*	Endothelial cells	Freshly isolated endothelial cells	NR	TUNEL assay (terminal deoxynucleotidyl transferase dUTP nick-end labeling; Roche)	Short-term exposure of endothelial cells to flavoring compounds used in tobacco products have adverse effects on endothelial cell phenotype	
	[46]	2001	To identify NtMEK2, a tobacco MAPKK. as an upstream kinase for both SIPK and WIPK	Animal	Rabbit model	Rabbit cells	NR	Antibody preparation and immunoblot analysis	MAPK cascade controls multiple defense responses against pathogen invasion	MAPK kinase, salicylic acid-induced protein kinase (SIPK) and wounding-induced protein kinase (WIPK)
	[47]	1998	To demonstrate that ungual cell wall-derived elicitor can activate SIPK in tobacco plants	Animal	Fungal cell model	Fungal cell wall elicitor	NR	Immunoprecipitation, immunoblot analysis, and immune-complex kinase assay	SIPK is involved in both disease resistance and response to wounding	SIPK (SA-induced protein kinase)
**Oxidative stress**	[51]	2009	To investigate oxidative and carcinogenic mechanisms of tobacco and synergistic action with other respirable particles in the respiratory system of smokers	Observational	Environmental exposure	Components of cigarettes	NR	Electron Paramagnetic Resonance (EPR) and spin-trapping techniques	Synergistic effects in the generation of HO•, through the Fenton reaction, with environmental respirable particles (asbestos fibers, coal dust, etc.)	Superoxide anion (O2•^–^) and hydroxyl (HO•) radicals
	[54]	2021	To determine the total antioxidant capacity, total oxidant status and oxidative stress index levels in the serum of active smokers, passive smokers and non-smokers	Observational	Biomarker-based	Humans	150	Spectro-photometric method using Rel Assay Diagnostics kit	OS levels in serum samples were significantly lower in non-smokers than smoker and past smoker groups	Antioxidants and total oxidant status (TOS)
	[55]	2016	To determine whether cigarette smoking affects (anti)oxidant status	Observational	Population-based	Humans	300	NA	Smoking as a risk factor for CAD is closely associated with increased oxidative stress, and the number of cigarettes smoked plays an important role in increasing the level of oxidative damage and reducing antioxidant defense	Concentration of oxidants (MDA and HP)
	[58]	2007	To determine the independent and combined impact of dietary intake and cigarette smoking on blood antioxidant capacity and oxidative stress	Observational	Cohort study	A sample of young smokers	28	ELISA procedure (Alpco Diagnostics, Salem, NH)	Cigarette smoking, particularly the number of years participating in this activity, may manifest in impaired antioxidant capacity and elevated oxidative stress independent of dietary intake	Plasma antioxidant reducing capacity (ARC; expressed in uric acid equivalents), serum trolox-equivalent antioxidant capacity (TEAC), whole blood total glutathione, plasma malondialdehyde (MDA), and plasma oxidized low density lipoprotein (oxLDL)
	[63]	2018	Whether the scavenging of mitochondrial H_2_O_2_ in transgenic mice expressing mitochondria-targeted catalase (mCAT) attenuates the development of cigarette smoke/angiotensin II-induced mitochondrial oxidative stress and hypertension	Animal	Mouse model	Transgenic mice expressing mitochondria-targeted catalase (mCAT) compared to wild-type mice	NR	Western blot experiment	Tobacco smoke and angiotensin II reduce the mitochondrial deacetylase sirtuin-3 level and cause hyperacetylation of a key mitochondrial antioxidant, SOD2, which promotes mitochondrial oxidative stress	SOD2
	[64]	2020	To investigate the regulatory mechanism underlying CS-induced hypoxia-inducible factor (HIF)-1α activation	*In vitro*	Human endothelial cells	Primary human endometrial stromal cells and an immortalized cell line (KC02-44D)	NR	Western blot analysis	CS extract (CSE) increased reactive oxygen species levels and stimulated HIF-1α protein stabilization in endometrial stromal cells	HI1-alpha expression
	[72]	2015	To assess the extent of oxidative damage induced by long-term cigarette smoke exposure	Animal	Rat model	Wistar rats	NR	Measurement of 8-OHdG in urine, lymphocytes, and lung tissue	Long-term cigarette smoke exposure can cause obvious damages of lung tissue in rats	Levels of ROS, 8-OHdG, and total antioxidant (T-AOC), expression of DNA repair enzymes, e.g. 8-oxyguaine DNA glycosylase (OGG1), and MutThomolog 1 (Oxidized Purine Nucleoside Triphosphatase, MTH1)
	[73]	2019	To clarify the direct effects of nicotine administration on the antioxidant defense system and lipid peroxidation	*In vitro*	Human endometrial cells	Human endometrial stromal primary cell	NR	Procedures of Fecondo and Augusteyn	Nicotine as a pro-oxidant affects the oxidative state of the endometrial cells	Glutathione (GSH) level, glutathione peroxidase (GPx), glutathione reductase (GR), and catalase (CAT) enzymes activity and higher levels of malondialdehyde (MDA)
	[75]	2021	To compare DNA damage marker localization, expression of DDR genes and expression of DNA repair genes in ectopic endometrial samples	Observational	Case-control	Women with and without endo-metriosis	66	RT2 Profiler PCR arrays	Alterations in the expression of DDR and DNA repair genes indirectly suggest that ectopic endometrium, as compared to its healthy counterpart, encounters DNA damage-inducing stimuli, either of higher strength or for longer duration in endometriosis	DNA damage response
	[76]	2018	Examined expression levels of genes pertaining to DNA DSB repair in patients with endometriosis to assess the potential effects on ovarian reserves	Observational	Case-control	Women with endo-metriosis	69	Immunohistochemistry	Expression of γ-H2AX in immunoassayed endometrial and ovarian tissue preparations was greater in the endometriosis group	DNA damage event
**Hormonal dysregulation**	[85]	2011	To investigate the relationship between cigarette smoking habits and endogenous sex hormone levels in postmenopausal women	Observational	Hormone analysis in post-menopause women	Post-menopausal women	2030	Non-fasting blood samples analyses	Cigarette smoking is associated with higher circulating levels of androgens, estrogens, 17-hydroxprogesterone, and SHBG	Androgens, estrogens, 17-hydroxprogesterone, and SHBG
	[88]	2018	To investigate the effect of nicotine on serum progesterone and estradiol levels as possible cause of abortion during first trimester of gestation	Animal	Rat model	Female Wistar rats	14	Enzyme-based immunoassay system	Significant decrease in serum progesterone and estradiol levels in the nicotine-treated group when compared to controls	Serum progesterone and estradiol levels
	[92]	2008	To test the hypothesis that cigarette smoking is associated with hot flushes through a mechanism involving androgen levels, progesterone levels, sex hormone binding globulin levels, or the ratio of androgens to estrogens	Observational	Self-reported hormonal data	Humans	628	Enzyme-linked immunosorbent assays (ELISA)	Cigarette smoking is associated with hot flushes through a mechanism that may not involve alterations in hormone levels or their ratios	Androgen and andro-stenedione levels
	[94]	2016	To investigate the effect of the non-aromatizable androgen dihydrotestosterone (DHT)	*In vitro*	Epithelial cell culture	Epithelial cells	NR	Real-time PCR	Significant DHT-dependent changes in the concentrations of mRNAs encoded by genes implicated in the regulation of the cell cycle	Non-aromatizable androgen dihydrotestosterone (DHT)
	[96]	2020	The potential sex steroid signal disrupting mechanisms of nicotine and cotinine	Computational	Molecular docking analysis	PubChem compound database	NR	PubChem compound database	Structural binding interactions of the tobacco alkaloid nicotine and its major metabolite cotinine with the sex-steroid nuclear receptors (nicotine and cotinine bind and interact with sex-steroid nuclear receptors and have potential to interfere in steroid hormone signaling resulting in reproductive dysfunction)	Estrogen receptor-α (ERα), ERβ, androgen receptor (AR), and progesterone receptor (PR)
	[99]	2002	To examine the circulating concentrations of IGF-1, IGFBP-3, and soluble ICAM-1	Observational	Hormonal study	Humans	20 smokers and 20 nonsmokers	ELISA (sICAM-1 Parameter Immunoassay, R&D Systems, Minneapolis, MN)	Soluble ICAM-1 concentrations were significantly increased in smokers, compared to non-smokers	ICAM-1; IGF-1; IGFBP-3
	[101]	2004	To examine whether nicotine inhibits the pulsatile gonadotropin-releasing hormone (GnRH) release, and whether this inhibition of GnRH release by nicotine is mediated by the GABA receptor system	Animal	Rat model	Wistar strain rats	NR	Roller tube culture	Nicotine stimulates GABA release, which then inhibits GnRH release through GABAA receptor system	GnRH, GABA
	[102]	1975	To evaluate the diagnostic and prognostic usefulness of the GnRH test, gonadotropin responses to iv GnRH	Observational	Human	Patients	82	Radio-immunoassay	GnRH reflect the readily releasable amount of LH which seems to correlate with previous exposure to endogenous GnRH	LH and FSH
**DNA damage**	[108]	2003	To evaluate whether mutagen sensitivity can predict the risk of endometriosis development	Observational	Genetic analysis	Patients	65 subjects and 46 control group	Cytogenetic analysis	Sensitivity to bleomycin-induced chromatid breaks in lymphocytes is associated with the risk of endometriosis development	Mutagen sensitivity of peripheral lymphocyte
	[110]	2008	To examine the levels and types of ROS that are produced in response to DNA damage	Animal	Rat model	Isogenic *S. cerevisiae* strains	NR		DNA damage-induced increase in intracellular ROS levels is a generalized stress response that is likely to function in various signaling pathways	ROS induced by DNA damage
	[116]	2020	To evaluate the direct effect of nicotine on the epigenome profiling	*In vitro*	Epigenetic study	Human endometrial stromal cells (HESC)	NR	Immunocytochemistry staining	Nicotine treatments reduced the average level of DNMTs gene expression	Genomic DNA methylation status and DNA methyl-transferases (DNMTs) gene expression
	[117]	2019	To investigate the impact of smoking on lung cells collected from bronchoalveolar lavage (BAL)	Observational	Genetic analysis in lung cells	Broncho-alveolar lavage samples from healthy volunteer	49	RNA sequencing	Tobacco smoke exposure epigenetically modifies BAL cells, possibly involving a continuous active demethylation and subsequent increased activity of inflammatory processes in the lungs	DNA methylation
	[118]	2014	To investigate if tobacco exposure can cause site-specific posttranslational histone modifications (PTMs)	*In vitro*	Histone modification study	Mouse and human bronchial epithelial cells (H292)	NR	Bottom-up mass spectrometry approach	Histone marks may play an important role in epigenetic state during the pathogenesis of smoking-induced chronic lung diseases	Histone H3 and histone H4
**Immune dysfunction**	[122]	2014	To test cigarette smoke extract on ovulation, oocyte morphology and ovarian gene expression associated with inhibition of oxidative stress	Animal	Mouse ovarian study	C57BL/6 mice	NR	Mice in the experimental group were administered a cigarette smoke extract (CSE) solution (2 mg/mL) orally daily, while the blank control group was given dimethylsulfoxide (DMSO). RNA extraction from ovaries	CSE group manifested a reduced diameter of zona pellucidafree oocyte (ZP-free OD) and a morphologically misshapen first polar body (PB)	Oocyte morphology and ovarian gene expression associated with inhibition of oxidative stress
	[123]	2003	To test the immunoregulatory effects of nicotine	*In vitro*	Immune cell study	Dendritic cells (DCs)	NR	ELISA kits	Nicotine can exert its immunosuppressive effects on immune surveillance through functional impairment of the DC system	Cytokines
	[124]	2020	To test the effects of smoking on inflammatory markers, innate and adaptive immune responses to bacterial and viral challenges and blood cell composition	Observational	Immune biomarker analysis	Plasma samples from heavy smokers	30	Luminex analysis and immunophenotyping	Smokers had lower NK cells and higher Tregs than controls, suggesting that smoking may reduce the ability to kill nascent tumor cells	CRP, fibrinogen, IL-6 and CEA levels
	[127]	2020	To evaluate the relationship between NK cell activity and urinary cotinine level	Observational	NK cell function analysis	Plasma by NK cells	12249	ELISA	NK cell activity was lower in current smokers	NK cell activity (IFN-gamma)
	[128]	2020	To detect the involvement of immune cells in the pathogenesis of endometriosis in patients with stable status or pelvic pain	Observational	Immune gene expression study	Blood was collected from patients with endo-metriosis	NR	Flow cytometry	SAMD9 and RGL2 expression levels were significantly upregulated in patients with pelvic pain	SAMD9 and RGL2 expression levels
	[133]	2017	Do cell adhesion molecules play a role in endometriosis, and can they be used as a biomarker for diagnosing endometriosis?	Observational	Cell adhesion molecules in serum	Serum of women	138	Quantitative real-time PCR	The mRNA levels of both VCAM-1 and ICAM-1 were higher in ectopic endometriotic lesions than in ectopic endometrium	Vascular cell adhesion molecule-1 (VCAM-1) and intercellular adhesion molecule-1 (ICAM-1)
	[135]	2012	To mimic, *in vitro*, the long-term exposure of human lung epithelium to smoke	*In vitro*	Lung epithelium model	Human lung adenocarcinoma cells (A549)	NR	Immunohistochemistry	Expression of Smad3 is lower in lung tumors of current smokers compared to that observed in never-smokers	Smad 3
	[145]	2006	To elucidate the role of angiogenic factors, we investigated *in vivo* whether blockade of vascular endothelial growth factor (VEGF), fibroblast growth factor (FGF), and platelet-derived growth factor (PDGF) affects angiogenesis of ectopic endometrium	Animal	Hamster angiogenesis study	Syrian golden hamsters	NR	Histology and immunohistochemistry	Vascularization of endometriotic lesions is not solely driven by VEGF, but depends on the crosstalk between VEGF, FGF and PDGF	VEGF, FGF and PDGF inhibitor SU6668
	[149]	2016	To elucidate pathophysiological processes *in vitro* and *in vivo* effects of tobacco extract on the transcription factor, hypoxia-inducible factor 1 (HIF-1)	*In vitro*	Hypoxia transcription factor study	A549 and BEAS-2B cells	NR	Immunoblot assays	CSE and CS induced HIF-1 activation *in vitro* and *in vivo*	HIF1-alpha expression
	[151]	2015	To investigate the expression of HIF-1a, HIF-2a, VEGF-A, PAR-1, and PAR-4 mRNA in lesions from patients with ovarian endometrioma (OMA) and deep infiltrating endometriosis (DIE)	Observational	HIF1-alpha and VEGF analysis	Ovarian endometrioma (OMA; n 1⁄4 16) or deep infiltrating endo-metriosis (DIE; n 1⁄4 11)	NR	Immunoblot assays	Ovarian endometrioma expresses high levels of HIF-1/2a, PAR-1/4, and VEGF-A. A positive correlation between the expression of HIF-1/2a and VEGF-A mRNA was observed in OMA	HIF-1a, HIF-2a, VEGF-A, PAR-1, and PAR-4 mRNA
	[152]	2017	To investigate whether autophagy was regulated by HIF-1α, as well as whether the effect of HIF-1α on cell migration and invasion is mediated through autophagy upregulation	Observational	Humans autophagy and invasion study	Human endometrial stromal cells (HESCs)	NR	Immunohistochemistry	HIF-1α promotes HESCs invasion and metastasis by upregulating autophagy	HIF-1α
	[154]	2014	To evaluate effects on remodeling and hyperreactivity face to tobacco expose	*In vitro*	Airway smooth muscle cell study	Canalicular-stage (18–20 wk gestational age) human fetal airway smooth muscle (fASM) cells	NR	Western blot analysis	These results demonstrate that cigarette smoke may enhance remodeling in developing human ASM through hyperplasia and ECM production	Signal-related kinase (ERK) and p38

Ref.: reference. NR: not reported.

Tobacco smoke components can activate immune cells in the pelvic cavity, including macrophages, neutrophils, and lymphocytes^27^. Activation of these immune cells triggers the secretion of proinflammatory mediators, such as interleukin 1beta (IL-1β), IL-6, IL-8, and tumor necrosis factor alpha (TNF-α)^28,29^. These cytokines play crucial roles in promoting inflammation, recruiting immune cells to the site of inflammation, and stimulating tissue remodeling processes^30^.

The nuclear factor-kappa B (NF-κB) pathway is a central regulator of inflammation^31^. The components in tobacco can activate the NF-κB pathway^32^, leading to the transcriptional upregulation of various pro-inflammatory genes^33^. NF-κB promotes the expression of cytokines, chemokines, adhesion molecules, and enzymes involved in the inflammatory response^34,35^. This sustained activation of NF-κB perpetuates the inflammatory environment in endometriosis^36,37^.

Tobacco smoke can stimulate the production of chemokines, such as IL-8 and monocyte chemoattractant protein-1 (MCP-1)^21,38^. These chemokines attract leucocytes, including neutrophils and macrophages, to endometriotic lesions^39-41^. The recruited immune cells contribute to the local inflammatory response and produce additional pro-inflammatory mediators, amplifying the inflammatory cascade^42^.

Tobacco can induce vascular permeability, leading to the leakage of plasma proteins and immune cells into the surrounding tissues^43^. This increased vascular permeability facilitates the infiltration of inflammatory cells into endometriotic lesions, exacerbating the inflammatory response^44^. Moreover, leakage of plasma proteins can further contribute to tissue inflammation and promote angiogenesis^45^.

Various signaling pathways involved in inflammation can be induced by tobacco, including the mitogen-activated protein kinase (MAPK) pathway^46,47^ and the Janus kinase/signal transducer and activator of transcription (JAK/STAT) pathway^48^. These pathways regulate the expression of pro-inflammatory genes and modulate immune cell function^49^. Activation of these pathways by tobacco smoke could contribute to the sustained inflammatory state in endometriosis^50^.

Thus, the inflammatory response could be triggered by tobacco smoke can lead to a dysregulated cytokine network in endometriosis. Cytokines, such as IL-1β, TNF-α, and IL-6, can induce the production of other inflammatory mediators and promote the activation of immune cells^29^. This cytokine crosstalk further amplifies the inflammatory cascade, perpetuating the chronic inflammatory environment in endometriotic lesions.

### Oxidative stress

Tobacco contains a variety of toxic chemicals and free radicals that can generate reactive oxygen species (ROS) when inhaled^51,52^ (Table 1). ROS, such as the superoxide anion (O2•^-^), hydrogen peroxide (H_2_O_2_), and the hydroxyl radical (OH•), are highly reactive molecules that can cause oxidative damage to cellular components, including lipids, proteins, and DNA^53^.

Oxidative stress induced by tobacco smoke overwhelms the body’s antioxidant defense mechanisms^52,54,55^. Antioxidants, such as glutathione, superoxide dismutase (SOD), and catalase, neutralize ROS and protect cells from oxidative damage^56^. However, tobacco smoke can deplete these antioxidants and impair their ability to counteract the excessive ROS production, leading to an imbalance between oxidative stress and the antioxidant capacity^57-59^.

ROS generated by tobacco can initiate lipid peroxidation, a process that damages cell membranes and disrupts their integrity^52^. Lipid peroxidation products, such as malondialdehyde (MDA) and 4-hydroxynonenal (4-HNE), can induce inflammation, impair cellular functions, and contribute to tissue damage^60^. In endometriosis, lipid peroxidation can affect the viability and function of endometrial cells, exacerbating the disease^61,62^.

Tobacco smoke-induced oxidative stress can impair mitochondrial function, including endometrial cells^63,64^. Mitochondria are a major source of ROS production, and their dysfunction can lead to increased ROS generation^65,66^. The compounds in tobacco can directly target mitochondria, disrupting their electron transport chain and impairing adenosine triphosphate (ATP) production^67,68^. Mitochondrial dysfunction further exacerbates oxidative stress^69,70^, perpetuating the cycle of oxidative damage and inflammation in endometriosis^71^.

ROS generated by tobacco can directly damage DNA in endometrial cells^72,73^. This DNA damage includes DNA strand breaks, base modifications, and DNA adduct formation^74^. Accumulated DNA damage can lead to genetic instability, mutations, and chromosomal aberrations in endometriotic lesions^75,76^. The compromised DNA repair mechanisms in endometriosis may exacerbate the impact of tobacco smoke-induced DNA damage on disease progression^77^.

Oxidative stress can activate various inflammatory signaling pathways in endometriosis^61,78,79^. ROS can stimulate the NF-κB pathway, leading to the production of pro-inflammatory cytokines and chemokines^80^. This activation of inflammatory pathways further amplifies the inflammatory response and contributes to the pathogenesis of endometriosis^81,82^.

Tobacco smoke-induced oxidative stress can result in the oxidation and modification of proteins and enzymes involved in cellular functions^52,57^. Oxidative modifications can disrupt protein structure and impair enzyme activity, changes that affect essential cellular processes^83^. In endometriosis, oxidative stress can target proteins and enzymes involved in inflammation, hormone signaling, and tissue remodeling, further contributing to disease progression^84^.

### Hormonal dysregulation

Tobacco has been associated with alterations in estrogen levels, which play a crucial role in the development and maintenance of endometriosis^7,15,85^ (Table 1). Smoking can decrease circulating estrogen levels by accelerating the metabolism and clearance of estrogen from the body^86^. This estrogen imbalance can disrupt the normal endocrine environment, potentially promoting the growth and survival of endometrial tissue outside the uterus^87^.

A decrease in progesterone levels has been linked by tobacco^88,89^. Progesterone is an important hormone that helps regulate the menstrual cycle and maintain the endometrium^87^. A decrease in progesterone levels may disrupt the balance between estrogen and progesterone, promoting the growth and proliferation of endometriotic lesions^90.^

Androgen hormone levels can be modulated by tobacco^91^. Smoking has been associated with increased androgen production and alterations in androgen metabolism^92^. These changes in androgen levels can affect the growth and survival of endometrial tissue outside the uterus^93,94^. Androgens, such as testosterone, can stimulate the growth of endometriotic lesions and contribute to the pathogenesis of endometriosis^95^.

Tobacco contains numerous chemicals that can interact with hormone receptors, including estrogen receptors (ERs) and progesterone receptors (PRs)^96^. These interactions can disrupt the normal signaling pathways regulated by these receptors. Altered receptor activation and signaling can affect gene expression patterns, leading to dysregulation of key genes involved in inflammation, cell proliferation, and tissue remodeling in endometriosis^97^.

The compounds in tobacco can interfere with hormone-related signaling pathways involved in endometriosis. For example, smoking has been shown to modulate the insulin-like growth factor (IGF) signaling pathway, which plays a role in cell growth and survival^98,99^. Dysregulation of hormone-related signaling pathways can contribute to the aberrant growth and survival of endometrial tissue in endometriosis^100^.

Tobacco can affect the hypothalamic-pituitary-gonadal axis by influencing the secretion and function of gonadotropin-releasing hormone (GnRH)^98,101^. GnRH is a key hormone that regulates the production of luteinizing hormone (LH) and follicle-stimulating hormone (FSH)^102^. Disruption of GnRH signaling by smoking can lead to imbalances in LH and FSH levels, which can impact ovarian function and the menstrual cycle, potentially contributing to endometriosis development and progression^98^.

Epigenetic modifications were associated with tobacco, including DNA methylation and histone modifications^103,104^. Epigenetic changes can alter gene expression patterns without altering the DNA sequence itself^105^. Smoking-induced epigenetic modifications can affect the expression of genes involved in hormonal regulation and contribute to hormonal dysregulation in endometriosis^104^.

### DNA damage and epigenetic modifications

Tobacco contains numerous harmful chemicals that can directly damage DNA^106^ (Table 1). These chemicals, such as PAHs, aromatic amines, and nitrosamines, can form DNA adducts and induce DNA strand breaks^107^. The DNA damage caused by tobacco smoke can lead to genetic alterations and chromosomal abnormalities in endometrial cells, potentially promoting the development and progression of endometriosis^8,108^.

The ROS generated by tobacco smoking can also cause oxidative damage to DNA^51^. ROS can react with DNA bases, leading to the formation of DNA adducts and base modifications^109^. Additionally, ROS can induce DNA strand breaks and impair DNA repair mechanisms^110^. The accumulation of oxidative DNA damage in endometrial cells can contribute to genomic instability and the pathogenesis of endometriosis^111^.

Tobacco smoking can interfere with DNA repair mechanisms in endometrial cells. The chemicals present in tobacco smoke can inhibit DNA repair enzymes, such as DNA polymerases and DNA repair proteins^112^. This impaired DNA repair capacity can lead to the persistence of DNA damage and genomic instability in endometriotic lesions, promoting disease progression^113^.

Epigenetic modifications refer to heritable changes in gene expression patterns without altering the DNA sequence itself^114^. Tobacco smoking has been associated with epigenetic modifications, including DNA methylation and histone modifications^103,104^. Smoking-induced epigenetic changes can alter the expression of genes involved in cellular processes such as inflammation, cell proliferation, and hormone signaling^115^. Thus, these modifications can contribute to the dysregulation of gene expression in endometrial cells and the pathogenesis of endometriosis.

Aberrant DNA methylation patterns in endometrial cells have been induced by tobacco smoking ^116^. DNA methylation is a common epigenetic modification that involves the addition of a methyl group to DNA molecules, typically leading to gene silencing^116^. Smoking-induced DNA methylation changes can affect the expression of genes involved in hormone metabolism, inflammation, and tissue remodeling, potentially promoting the development and progression of endometriosis^104,117^.

Tobacco smoking can also influence histone modifications, which regulate the accessibility of DNA to transcription factors and other proteins involved in gene expression^118^. Smoking-induced histone modifications can alter the structure of chromatin and affect the expression of genes implicated in endometriosis^104,119^. These modifications can lead to dysregulated gene expression patterns and contribute to the molecular and cellular changes associated with the disease.

Tobacco smoking-induced DNA damage and epigenetic modifications can potentially have transgenerational effects on offspring^120^. Smoking-related alterations in sperm and egg cells can lead to inherited epigenetic changes that may influence the susceptibility to endometriosis in future generations^121,122^. These transgenerational effects highlight the long-lasting impact of tobacco smoking on the molecular pathways involved in endometriosis.

### Immune dysfunction

Tobacco can modulate the immune system, leading to dysregulation of immune cells and molecules involved in the pathogenesis of endometriosis^27^ (Table 1). Smoking can suppress the activity of immune cells, such as natural killer (NK) cells, macrophages, and T cells, reducing their ability to eliminate endometrial cells outside the uterus^8,123^. This impaired immune response allows the survival and proliferation of ectopic endometrial tissue, contributing to the development of endometriosis.

Tobacco may disrupt the production and balance of cytokines, which are important immune signaling molecules^21^. Smoking has been associated with increased production of pro-inflammatory cytokines, such as IL-6 and TNF-α, and decreased production of anti-inflammatory cytokines, such as IL-10^124^. This imbalance in cytokine production can contribute to chronic inflammation and tissue damage in endometriosis^125^.

A chronic inflammatory state in the body is induced by tobacco smoking, characterized by elevated levels of inflammatory markers and immune cells^124^. Smoking-related inflammation can promote the recruitment of immune cells to endometriotic lesions and exacerbate tissue inflammation^22^. This persistent inflammatory response can contribute to the growth, invasion, and persistence of endometriotic lesions^126^.

Tobacco smoking can affect the function of immune cells involved in endometriosis. For example, smoking can impair the cytotoxic activity of NK cells, which play a crucial role in eliminating abnormal cells, including endometrial cells^127,128^. Smoking-related alterations in immune cell function can compromise the surveillance and clearance of endometrial cells outside the uterus, contributing to the establishment and progression of endometriosis^129^.

Immune tolerance refers to the ability of the immune system to recognize and tolerate selft-issues^130^. In endometriosis, there is a breakdown in immune tolerance, allowing ectopic endometrial tissue to survive and evade immune surveillance^126,131^. Tobacco smoking can further disrupt immune tolerance mechanisms, leading to an aberrant immune response against endometrial cells and perpetuating the immune dysregulation observed in endometriosis21,27,104.

The compounds found in tobacco smoke can modulate the expression and function of cellular adhesion molecules involved in immune cell trafficking and tissue inflammation. Smoking-induced alterations in adhesion molecules, such as intercellular adhesion molecule-1 (ICAM-1) and vascular cell adhesion molecule-1 (VCAM-1), can recruit immune cells to endometriotic lesions and contribute to the inflammatory process^132,133^.

Tobacco smoking can impair wound healing and tissue repair processes, which are essential for the resolution of inflammation and the restoration of tissue integrity^134^. Smoking-related factors can interfere with the production and activity of growth factors, such as transforming growth factor beta (TGF-β), which play a critical role in tissue repair^135^. Impaired wound healing can perpetuate the inflammatory response and contribute to the persistence and progression of endometriotic lesions^136^.

### Angiogenesis

Angiogenesis refers to the formation of new blood vessels from pre-existing ones^137^ (Table 1). In endometriosis, angiogenesis plays a crucial role in the establishment and growth of ectopic endometrial tissue^138^. Tobacco smoking has been linked to increased angiogenesis^139-141^, which can contribute to the progression and persistence of endometriotic lesions.

Tobacco smoke contains various chemicals that can promote angiogenesis. For example, nicotine, a key component of tobacco, has been shown to stimulate the release of pro-angiogenic factors, such as vascular endothelial growth factor (VEGF)^142^ and basic fibroblast growth factor (bFGF)^143^. These factors can enhance the formation of new blood vessels, providing a blood supply to endometriotic lesions and supporting their growth^144,145^.

In addition to promoting angiogenesis, tobacco smoking can disrupt the balance of angiogenesis inhibitors ^141^. Endostatin, thrombospondin-1 (TSP-1), and angiostatin are examples of naturally occurring substances that inhibit blood vessel formation^146,147^. Smoking-related factors could interfere with the production and function of these angiogenesis inhibitors, thus allowing angiogenesis to proceed unchecked in endometriosis.

Inflammatory cells and cytokines present in the endometriotic micro-environment can promote the production of pro-angiogenic factors, which contribute to neovascularization^137,148^. These newly formed blood vessels provide nutrients and oxygen to endometriotic lesions, facilitating their survival and growth.

Tobacco can induce hypoxic conditions in tissues due to decreased oxygen availability^149,150^. Hypoxia is a potent stimulator of angiogenesis, as it triggers the release of hypoxia-inducible factors (HIFs)^137^. These proteins promote the expression of VEGF and other pro-angiogenic factors, facilitating the formation of new blood vessels in the hypoxic environment of endometriotic lesions^151,152^.

Tobacco smoking can disrupt the remodeling of the extracellular matrix (ECM), which is essential for angiogenesis^153^. The ECM provides structural support for blood vessels and influences their formation and stability^154^. Smoking-related factors can affect the production and degradation of ECM components^155^, leading to an imbalance in ECM remodeling and promoting angiogenesis in endometriosis.

Angiogenesis supports the growth of endometriotic lesions and facilitates their invasion into surrounding tissues. The newly formed blood vessels provide a pathway for the migration of endometrial cells, enabling them to establish new lesions and to expand the disease. Therefore, smoking-induced angiogenesis can contribute to the invasive and metastatic behavior of endometriosis.

### Limitations

While this narrative review provides a synthesis of existing literature on the relationship between tobacco smoking and endometriosis, several limitations must be acknowledged. First, most of the included studies rely on observational data, which inherently limits causal inference (Table 2). Although emerging evidence suggests a potential link between smoking and endometriosis, confounding factors such as genetic predisposition, environmental exposures, and lifestyle factors may influence the observed associations. Second, self-reported smoking status, a common data collection method in epidemiological studies, may introduce recall bias and misclassification. Individuals may underreport or overestimate their smoking behavior, leading to potential misinterpretation of results. Additionally, differences in study designs, population characteristics, and exposure definitions contribute to heterogeneity across studies, making direct comparisons challenging.

**Table:** Table 2. Risk of bias assessment of studies included, based on Newcastle-Ottawa Scale (Nos) for observational studies, Risk of Bias 2 (RoB2) for randomized trials, and SYRCLE’s Risk of Bias tool for animal studies

*Ref.*	*Risk of bias*
[23]	Moderate (*in vitro*, lacks systemic physiological context)
[24]	High (self-reported exposure, potential confounding)
[25]	Moderate (biomarker-based, but cross-sectional design)
[32]	Moderate (controlled setting, but animal model limitations)
[39]	High (small sample size, selection bias)
[40]	Moderate (no clinical correlation)
[43]	Moderate (limited real-world application)
[46]	Moderate (animal model not directly transferable)
[47]	Moderate (animal model not directly transferable)
[51]	Moderate (experimental setting, lacks direct application to humans)
[54]	Moderate (used objective biomarkers, but cross-sectional)
[55]	High (no control for confounders, limited sample size)
[58]	Moderate (controlled for diet but self-reported smoking)
[63]	Moderate (animal model, potential extrapolation issues)
[64]	Moderate (cell-based, lacks human validation)
[72]	Moderate (measured oxidative stress markers, no human validation)
[73]	Moderate (*in vitro*, lacks physiological relevance)
[75]	High (small sample, lacks confounder control)
[76]	High (observational, small sample, no randomization)
[85]	Moderate (association study, no causal inference)
[88]	Moderate (animal model, indirect human relevance)
[92]	High (self-reported symptoms, potential recall bias)
[94]	Moderate (lacks systemic validation)
[96]	Low (computational, needs experimental validation)
[99]	Moderate (small cohort, observational limitations)
[101]	Moderate (animal model, indirect human relevance)
[102]	High (small sample, endocrine condition variability)
[108]	High (genetic study, lacks environmental control)
[110]	Moderate (oxidative stress analysis, extrapolation issues)
[116]	Moderate (epigenetic changes, lacks longitudinal data)
[117]	Moderate (DNA methylation, lacks direct causality)
[118]	Moderate (histone modifications, lacks human correlation)
[122]	Moderate (ovarian morphology, unclear generalizability)
[123]	Moderate (immune study, lacks functional validation)
[124]	High (observational, lacks control group)
[127]	High (NK activity, lacks exposure quantification)
[128]	High (immune markers, lacks comprehensive control)
[133]	Moderate (cell adhesion markers, lacks validation)
[135]	Moderate (lung epithelium, not reproductive model)
[145]	Moderate (vascular markers, lacks systemic relevance)
[149]	Moderate (oxidative markers, lacks intervention)
[151]	Moderate (HIF-1α activation, lacks real-world correlation)
[152]	Moderate (autophagy markers, lacks systemic insight)
[154]	Moderate (cell model, lacks *in vivo* validation)

Ref.: reference.

The biological mechanisms linking tobacco smoking to endometriosis remain complex and incompletely understood. While this narrative review highlights several molecular pathways, such as inflammation, oxidative stress, hormonal dysregulation, and epigenetic modifications, causality cannot be definitively established. Further experimental and longitudinal studies are needed to clarify these mechanisms. Finally, while this narrative review provides an overview of the evidence, a systematic review with a comprehensive search strategy, critical appraisal of included studies, and synthesis of findings, would have provided a more conclusive evidence base and hence would be warranted.

## CONCLUSION

This review highlights the growing body of evidence linking tobacco smoking to the pathogenesis of endometriosis through multiple biological mechanisms, including chronic inflammation, oxidative stress, hormonal dysregulation, immune dysfunction, and epigenetic modifications. While early studies provided conflicting results, recent large-scale epidemiological data and mechanistic insights suggest that smoking is not only a risk factor for endometriosis but may also exacerbate its severity and progression. The detrimental effects of tobacco on endometrial tissue underscore the broader impact of smoking on women’s reproductive health. This result highlights once again the specific impact of tobacco consumption on women’s health, and adds endometriosis to an already long list (hormone-dependent^156^, infertility^157^, cardiovascular pathologies^158^, for example). Despite these findings, several critical gaps remain. The causality between smoking and endometriosis has yet to be definitively established, necessitating prospective cohort studies with robust control for confounding factors. Future research should also integrate omics approaches, such as transcriptomics, proteomics, and metabolomics, to unravel the molecular pathways underlying the link between tobacco exposure and endometriosis. Additionally, identifying biomarkers of tobacco-induced endometriotic changes could facilitate early diagnosis and risk stratification. From a clinical and public health perspective, these findings reinforce the need for targeted smoking cessation interventions, particularly for women at risk of or diagnosed with endometriosis. Healthcare professionals should incorporate smoking history assessments into routine gynecological care and emphasize the role of smoking in disease progression. Public health policies should also focus on prevention strategies to reduce smoking rates among young women, thereby mitigating a modifiable risk factor for endometriosis and improving reproductive health outcomes.

## Data Availability

Data sharing is not applicable to this article as no new data were created.
